# The relationship between physical activity and music performance anxiety among university music students: a chain mediation model

**DOI:** 10.3389/fpsyg.2026.1895317

**Published:** 2026-08-31

**Authors:** Shuchang Liu, Xingyi Li, Jiangtao Han, Shuting Liu

**Affiliations:** 1 Department of Education, Izmail State University of Humanities, Izmail, Ukraine; 2 Department of Sport and Health, Shinhan University, Uijeongbu, Gyeonggi Province, Republic of Korea; 3 School of Social Work and Health Management, Xihua University, Chengdu, China; 4 Department of Physical Education, Sejong University, Seoul, Republic of Korea

**Keywords:** chain mediation, cognitive reappraisal, music performance anxiety, physical activity, psychological resilience

## Abstract

**Introduction:**

Music performance anxiety (MPA) is a prominent psychological concern among university music students. Although physical activity has been associated with lower levels of anxiety, its association with music performance anxiety has received limited empirical attention. This study examined the association between physical activity and music performance anxiety among university music students, with particular attention to the mediating roles of psychological resilience and cognitive reappraisal.

**Methods:**

A convenience sample of 1,236 music students was recruited from multiple universities in China. Participants completed measures of physical activity, psychological resilience, cognitive reappraisal, and music performance anxiety. Mediation and serial mediation analyses were conducted using Model 6 of the SPSS PROCESS macro, with bootstrap estimation based on 5,000 resamples.

**Results:**

In the total-effect model, physical activity was significantly and negatively associated with music performance anxiety (*B* = −0.574, *p* < 0.01). After psychological resilience and cognitive reappraisal were included in the model, the direct association remained significant but was attenuated (*B* = −0.328, *p* < 0.01). Bootstrap analyses indicated significant indirect effects through psychological resilience (indirect effect = −0.132, Boot SE = 0.018, 95% CI [−0.170, −0.098]) and cognitive reappraisal (indirect effect = −0.081, Boot SE = 0.014, 95% CI [−0.109, −0.056]). The serial indirect effect through psychological resilience and cognitive reappraisal was also significant (indirect effect = −0.033, Boot SE = 0.006, 95% CI [−0.045, −0.023]).

**Conclusion:**

These findings extend understanding of the associations among physical activity, psychological resilience, cognitive reappraisal, and music performance anxiety. Psychological resilience and cognitive reappraisal may represent relevant psychological factors in the association between physical activity and music performance anxiety, providing a preliminary basis for future longitudinal and intervention research and for educational practices intended to support music students' psychological well-being.

## 1 Introduction

Music performance anxiety (MPA) refers to a marked and persistent anxiety response experienced in music performance settings and may manifest across emotional, cognitive, physiological, and behavioral domains (Kenny, 2010). It is common among both music students and professional performers and is associated with physiological tension, negative worry, avoidance tendencies, poorer mental health, and impaired professional development (Fernholz et al., 2019). At higher levels, MPA may also reduce music students' willingness to participate in performance activities and negatively affect their longer-term career development (Wang and Yang, 2024). MPA therefore represents an important psychological challenge for university music students.

Given its prevalence and adverse consequences, a range of interventions has been developed to address MPA, including cognitive-behavioral therapy, exposure therapy, biofeedback, beta-blockers, relaxation training, and mindfulness-based approaches (Burin and Osorio, 2016; Fernholz et al., 2019; Gómez-López and Sánchez-Cabrero, 2023). However, existing approaches vary considerably in their targets and effects, and some primarily focus on alleviating physiological tension or short-term anxiety responses rather than addressing the broader cognitive and emotional processes involved in MPA. Earlier work has similarly noted that interventions focused mainly on tension reduction may not fully address the cognitive and emotional processes underlying performance anxiety (Taborsky, 2007). Moreover, despite the prevalence of MPA, relatively few music students and professional performers actively seek professional help (Kaspersen and Götestam, 2002; Kinney et al., 2025). Some psychological and multimodal interventions also require trained professionals, substantial time, or specialized resources, which may limit their widespread implementation in higher education settings. Therefore, identifying accessible, scalable, and psychologically beneficial approaches that can be more readily integrated into university settings remains important.

Physical activity may represent one such modifiable behavioral factor because of its accessibility and established mental-health benefits. Regular physical activity has been associated with lower anxiety and better psychological well-being in broader populations (Biddle and Asare, 2011; Rebar et al., 2015), and emerging research has examined its relevance to MPA. Rocha et al. (2014) found that physically active music students reported lower MPA than their inactive peers, whereas Hernández-Dionis et al. (2025) found no significant association among higher music education students. Using an experimental approach, Blasco-Lafarga et al. (2022) reported reduced performance anxiety following acute high-intensity exercise in a small sample of wind musicians. Although these findings suggest that physical activity may be relevant to MPA, the evidence remains limited and differs considerably in study design, physical activity exposure, and sample characteristics. More importantly, previous research has provided little insight into the psychological processes that may underlie this relationship.

One psychological factor that may help explain this association is psychological resilience. Resilience reflects the capacity to adapt successfully when confronted with stress or adversity (Connor and Davidson, 2003) and has been used to explain why some individuals maintain relatively stable psychological functioning under sustained pressure (Bonanno, 2008; Kalisch et al., 2015). Physical activity has been positively associated with resilience, and meta-analytic evidence suggests that resilience may help explain relationships between physical activity and mental-health outcomes (Lin et al., 2024). In music performance contexts, higher resilience has also been associated with lower MPA (Du et al., 2025). Resilience may therefore help explain why physically active music students are better able to adapt to performance-related stress.

From the perspective of emotion regulation, cognitive reappraisal is also relevant because music performance anxiety is shaped not only by performance demands and physiological arousal, but also by how performers interpret these experiences. Cognitive reappraisal is an antecedent-focused emotion-regulation strategy through which individuals reinterpret the meaning of a situation in order to alter its emotional impact (Gross and John, 2003). In evaluative situations, for example, interpreting pre-performance arousal as excitement rather than threat may lead to more adaptive emotional responses (Brooks, 2014). Existing research has also linked habitual physical activity with greater use of cognitive reappraisal and with related cognitive-control processes (Giles et al., 2017; Perchtold-Stefan et al., 2020). These findings suggest that cognitive reappraisal may help explain how music students interpret and regulate emotional responses to nervousness, physiological arousal, mistakes, and external evaluation in performance settings.

Taken together, psychological resilience and cognitive reappraisal represent two distinct psychological processes that may be relevant to the relationship between physical activity and music performance anxiety. Psychological resilience concerns individuals' broader capacity to adapt to and recover from performance-related stress, whereas cognitive reappraisal concerns how they interpret and regulate the emotional impact of specific performance experiences. Although both factors have received increasing attention in related research, they have rarely been examined together in the context of music performance anxiety. It therefore remains unclear whether they may help explain the relationship between physical activity and music performance anxiety among university music students.

Accordingly, the present study examined the relationship between physical activity and music performance anxiety and further tested the roles of psychological resilience and cognitive reappraisal within a chain mediation model. By considering these factors within the same analytical framework, the study may contribute to a more detailed understanding of how physical activity, psychological resilience, cognitive reappraisal, and music performance anxiety are related among university music students. The findings may also provide a preliminary empirical basis for developing more comprehensive approaches to supporting music students' psychological well-being and managing performance-related anxiety in higher education settings.

## 2 Hypothesis development

### 2.1 Physical activity and music performance anxiety

Conservation of Resources (COR) theory posits that individuals seek to acquire, maintain, and protect valued resources when facing stress; resource loss intensifies stress responses, whereas resource gain can strengthen resistance to stress (Hobfoll, 1989). Music performance is a highly demanding context that can consume emotional and cognitive resources and increase physiological and psychological strain. Within the COR framework, behaviors that help restore or strengthen such resources may therefore be associated with lower anxiety responses.

Physical activity is regarded as a typical resource-gaining behavior. A substantial body of research has shown that regular physical activity not only enhances emotion regulation capacity (Bernstein and McNally, 2017), but also alleviates stress and strengthens self-efficacy (McAuley and Blissmer, 2000; Puett et al., 2014), thereby generating resource gains across multiple levels. From the perspective of COR theory, physical activity may help music students accumulate the various resources needed to cope with highly stressful performance situations. Moderate- to high-intensity physical activity, in particular, may relieve the stress associated with performance preparation, promote emotional recovery, and enhance adaptation to stage-related pressure (Blasco-Lafarga et al., 2022). Low-intensity mind-body activities such as yoga have likewise been shown to effectively reduce performance anxiety (Khalsa et al., 2013), further suggesting that different forms of physical activity may exert beneficial effects through resource replenishment mechanisms. Accordingly, the following hypothesis was proposed:

H1: Physical activity is significantly negatively associated with music performance anxiety among university music students.

### 2.2 The mediating role of psychological resilience

Within the COR framework, psychological resilience can be regarded as an important resource that enables individuals to maintain or regain adaptive functioning under stress. This capacity is particularly relevant to music performance, which places substantial emotional and cognitive demands on performers and may deplete their coping resources (Kenny, 2011; McPherson and McCormick, 2006). Regular physical activity may contribute to resilience by promoting positive emotional experiences, strengthening self-efficacy, improving psychological functioning, and facilitating recovery from stress (Biddle and Asare, 2011; Rebar et al., 2015). These resource gains may reinforce further psychological adaptation and thereby strengthen resilience (Chen et al., 2015; Hobfoll et al., 2018). In turn, greater resilience has been associated with lower music performance anxiety, partly through enhanced perceived control and reduced negative rumination (Du et al., 2025). Psychological resilience may therefore mediate the relationship between physical activity and music performance anxiety. This suggests that resilience may not only be associated with individuals' emotional responses under performance pressure, but may also serve as an important psychological correlate in the relationship between physical activity and music performance anxiety. On this basis, the following hypothesis was proposed:

H2: Resilience mediates the relationship between physical activity and music performance anxiety among university music students.

### 2.3 The mediating role of cognitive reappraisal

Music performance anxiety is influenced not only by performance demands but also by how performers interpret stressful situations and physiological arousal (Kenny, 2011). Cognitive reappraisal is an antecedent-focused emotion-regulation strategy through which individuals alter the emotional impact of a situation by changing its interpretation (Gross and John, 2003). In music performance, viewing physiological arousal as evidence of impending failure may intensify anxiety, whereas interpreting it as a normal part of performance preparation may reduce perceived threat (Papageorgi et al., 2013; Brooks, 2014). Physical activity may facilitate cognitive reappraisal by supporting emotional stability, executive functioning, cognitive flexibility, and self-efficacy, all of which may promote less threatening interpretations of stressful experiences (Hillman et al., 2008; Bernstein and McNally, 2017; Mandolesi et al., 2018; Perchtold-Stefan et al., 2020; Zhang et al., 2019). Cognitive reappraisal may therefore mediate the relationship between physical activity and music performance anxiety. On this basis, the following hypothesis was proposed:

H3: Cognitive reappraisal mediates the relationship between physical activity and music performance anxiety among university music students.

### 2.4 The chain mediating roles of psychological resilience and cognitive reappraisal

Conservation of Resources theory posits that individuals' capacity to cope with stress depends substantially on the psychological resources they are able to acquire, retain, and mobilize. Existing resources not only help individuals protect themselves against further resource loss, but may also facilitate the acquisition and effective use of additional resources, thereby generating an ongoing process of resource gain (Hobfoll, 1989; Hobfoll et al., 2018). From this perspective, relatively stable psychological resources available to individuals under stress may provide a foundation for the subsequent use of more specific adaptive coping and regulatory strategies (Chen et al., 2015). Psychological resilience and cognitive reappraisal can be understood as operating at different stages of this resource process. Psychological resilience reflects the capacity to maintain or regain adaptive functioning in the face of stress or adversity, whereas cognitive reappraisal is a more specific emotion-regulation strategy through which individuals modify the emotional impact of a stressful situation by changing how it is interpreted (Connor and Davidson, 2003; Gross and John, 2003). Although previous research has reported a relatively consistent association between resilience and cognitive reappraisal (Stover et al., 2024), this association alone does not establish their temporal ordering. In the present study, psychological resilience was positioned before cognitive reappraisal on the basis of the different functions that the two constructs may serve in the stress-response process. Psychological resilience more broadly represents a resource base that supports psychological stability and adaptation under stress, whereas cognitive reappraisal reflects the use of cognitive regulation to reinterpret experiences within a specific stressful situation.

Existing evidence provides some support for this proposed ordering. Individuals with higher levels of psychological resilience generally demonstrate greater emotional stability, cognitive flexibility, and self-regulatory capacity, and are less likely to appraise stressful situations as overwhelming or unmanageable threats (Tugade and Fredrickson, 2004; Kalisch et al., 2015). This relatively stable psychological state may preserve greater cognitive and emotional regulatory capacity under pressure, thereby facilitating more flexible and adaptive reinterpretation of the immediate situation. In music performance contexts, students are required to manage multiple sources of pressure simultaneously, including physiological arousal, the possibility of mistakes, and external evaluation. Greater psychological resilience may help them maintain a comparatively stable psychological state under these conditions, making it easier to use cognitive reappraisal to interpret nervousness, physiological responses, and evaluative pressure in less threatening ways. On the basis of this theoretical reasoning and the available empirical evidence, the following hypothesis was proposed:

H4: Resilience and cognitive reappraisal jointly play a chain mediating role in the relationship between physical activity and music performance anxiety among university music students.

## 3 Current study

**Figure 1:**
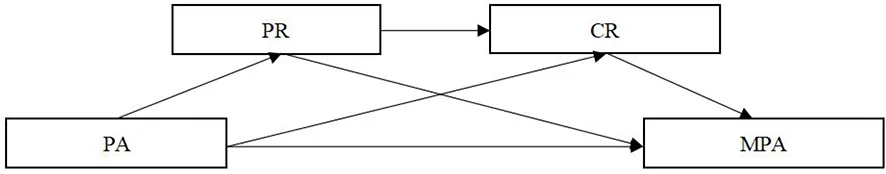
Concept model. PA, physical activity; PR, Psychological resilience; CR, cognitive reappraisal; MPA, music performance anxiety.

In summary, the present study aimed to examine the relationship between physical activity and music performance anxiety among university students, and to further explore the potential roles of resilience and cognitive reappraisal in this association. Focusing on these two key psychological resources, this study developed a chain mediation model (see Figure 1) to examine the pattern of associations among physical activity, resilience, cognitive reappraisal, and music performance anxiety. On this basis, the following hypotheses were proposed:

(1) H1: Physical activity is significantly negatively associated with music performance anxiety among university music students.
(2) H2: Psychological resilience mediates the relationship between physical activity and music performance anxiety among university music students.
(3) H3: Cognitive reappraisal mediates the relationship between physical activity and music performance anxiety among university music students.
(4) H4: Psychological resilience and cognitive reappraisal jointly play a chain mediating role in the relationship between physical activity and music performance anxiety among university music students.

## 4 Method

### 4.1 Participants

This study used convenience sampling to recruit undergraduate students majoring in music from multiple universities in Jiangsu, Guangxi, Shandong, and other regions of China. The study was conducted in accordance with the Declaration of Helsinki and was approved by the Human Research Ethics Committee of Xihua University (approval no. XHLL2025-074x). With the assistance of teachers, the research team contacted eligible students and provided them with information about the study purpose, survey content, and participation requirements. Questionnaires were distributed through a combination of online platforms and in-person administration. Before completing the questionnaire, all participants were provided with a detailed explanation of the study and read the informed consent form. Participation was entirely voluntary, and participants were informed that they could withdraw from the study at any stage without any adverse consequences. All questionnaires were completed anonymously, and the collected data were used solely for the statistical analyses of the present study to protect participants' privacy and data confidentiality.

A total of 1,368 questionnaires were distributed. During data screening, 132 questionnaires (9.65%) were excluded because they exhibited long strings of identical responses across questionnaire items or contained excessive missing responses on key study variables. Ultimately, 1,236 valid questionnaires were retained for analysis, corresponding to a valid response rate of 90.35%. The final sample comprised 632 male students (51.13%) and 604 female students (48.87%), with participants ranging in age from 18 to 24 years. Regarding academic year, 320 participants were freshmen (25.89%), 310 were sophomores (25.08%), 305 were juniors (24.68%), and 301 were seniors (24.35%). In terms of music-study experience, 180 participants had studied music for fewer than 3 years (14.56%), 330 for 3–5 years (26.70%), 360 for 6–8 years (29.13%), 270 for 9–11 years (21.84%), and 96 for 12 years or more (7.77%).

To evaluate the adequacy of the achieved sample size, a sensitivity power analysis was conducted using G^*^Power 3.1. With a total sample of 1,236 participants, an alpha level of 0.05, and statistical power of 0.95, the analysis indicated that the study was capable of detecting an effect size as small as *f*^2^ = 0.018. These results indicated that the achieved sample provided sensitivity to detect small effects in the planned regression models.

### 4.2 Instruments

#### 4.2.1 Music performance anxiety

Music performance anxiety was measured using the Chinese-language Stage Music Performance Anxiety Inventory developed and validated by Su et al. (2017) among Chinese-speaking music students. The instrument is designed to assess anxiety experienced in actual performance situations and comprises two dimensions: physiological anxiety and stage-related worry. It contains 14 items rated on a 7-point Likert scale ranging from 0 (never) to 6 (always). Item scores are summed to produce a total score ranging from 0 to 84, with higher scores indicating higher levels of music performance anxiety. The scale has been used in the Chinese cultural context and has demonstrated good reliability and validity in related research (Du et al., 2025). In the present study, Cronbach's alpha for the total scale was 0.908. To further examine the construct validity of the measure, confirmatory factor analysis (CFA) was conducted. The results indicated an acceptable model fit: χ^2^(76) = 420.21, *p* < 0.001, comparative fit index (CFI) = 0.949, Tucker–Lewis index (TLI) = 0.939, root mean square error of approximation (RMSEA) = 0.061, and standardized root mean square residual (SRMR) = 0.036. Overall, the major fit indices met commonly recommended criteria for good or acceptable model fit, providing support for the validity of the measure.

#### 4.2.2 Psychological resilience

Psychological resilience was assessed using the Chinese version of the 10-item Connor–Davidson Resilience Scale (CD-RISC-10). The CD-RISC-10 was developed and validated by Campbell-Sills and Stein (2007) as an abbreviated version of the original 25-item CD-RISC developed by Connor and Davidson (2003). The Chinese version has demonstrated good psychometric properties in Chinese populations (Cheng et al., 2020). The scale assesses individuals' capacity to adapt to and recover from adversity, stress, challenges, and difficult circumstances. It contains 10 items rated on a 5-point Likert scale ranging from 0 (not true at all) to 4 (true nearly all the time). Item scores are summed to produce a total score ranging from 0 to 40, with higher scores indicating greater psychological resilience. In the present study, Cronbach's alpha for the scale was 0.907. To further examine the construct validity of the measure, confirmatory factor analysis (CFA) was conducted. The results indicated a good model fit: χ^2^(35) = 146.99, *p* < 0.001, CFI = 0.980, TLI = 0.975, RMSEA = 0.051, and SRMR = 0.023. Overall, the major fit indices met commonly recommended criteria for good or acceptable model fit, providing support for the validity of the measure.

#### 4.2.3 Cognitive reappraisal

Cognitive reappraisal was measured using the cognitive reappraisal subscale of the Emotion Regulation Questionnaire developed by Gross and John (2003). The questionnaire was translated and adapted for use in China by Wang et al. (2007) and has demonstrated good reliability and validity in the Chinese cultural context. The cognitive reappraisal subscale consists of 6 items rated on a 7-point Likert scale ranging from 1 (strongly disagree) to 7 (strongly agree). Item scores are summed to produce a total score ranging from 6 to 42, with higher scores indicating a greater tendency to use cognitive reappraisal as an emotion regulation strategy. In the present study, Cronbach's alpha for the subscale was 0.808. To further examine the construct validity of the measure, confirmatory factor analysis (CFA) was conducted. The results indicated a good model fit: χ^2^(9) = 46.21, *p* < 0.001, CFI = 0.981, TLI = 0.968, RMSEA = 0.058, and SRMR = 0.024. Overall, the major fit indices met commonly recommended criteria for good or acceptable model fit, providing support for the validity of the measure.

#### 4.2.4 Physical activity

Physical activity was measured using the Physical Activity Rating Scale developed by the Japanese scholar Kimio Hashimoto and later revised by the Chinese scholar Liang Deqing (Hashimoto, 1990; Liang, 1994). This scale was culturally adapted to the Chinese context and to the realities of university students, and is now widely used among Chinese college populations. The PARS-3 assesses individuals' level of physical activity across three dimensions: exercise intensity, exercise frequency, and exercise duration, each rated on a 5-point Likert scale. According to the scoring instructions, total physical activity is calculated using the formula: physical activity score = exercise frequency × (exercise duration – 1) × exercise intensity. Total scores range from 0 to 100, with higher scores indicating higher levels of physical activity. Previous studies have shown that the applicability of this scale among Chinese university students has been extensively validated (Mu et al., 2024; Zhang et al., 2025).

### 4.3 Control variable

Gender, age, academic year, and years of musical training were included as control variables in the present study. Their selection was based primarily on previous research examining demographic characteristics and music-training backgrounds in relation to music performance anxiety. Existing evidence suggests that music performance anxiety may vary according to individual demographic characteristics, with gender and age among the factors most frequently considered. Sokoli et al. (2022) found that female music students reported higher levels of pre-performance anxiety and catastrophizing, while age was also significantly associated with several indicators of music performance anxiety. A systematic review similarly identified gender and age as important sources of individual variation in music performance anxiety, although the direction and strength of the association between age and music performance anxiety may differ according to the age range of the sample and performers' stage of professional development (Fernholz et al., 2019). Gender and age were therefore included as demographic covariates. Academic year and years of musical training were also included to account, at least partially, for differences in students' stage of professional training and accumulated musical experience. In a study of students in higher music education, Casanova et al. (2018) found that differences in music performance anxiety across academic years varied according to instrument type, with some senior students reporting higher levels of anxiety than their junior counterparts. This finding suggests that students' stage of professional training and the performance demands they encounter may be associated with music performance anxiety. Years of musical training were additionally controlled to reduce the potential influence of differences in accumulated musical experience on the relationships among the principal study variables (Sokoli et al., 2022). On the basis of this evidence, gender, age, academic year, and years of musical training were entered as covariates in the analyses to account for potential variation associated with demographic and training-related characteristics.

### 4.4 Data analysis

The data analysis comprised six main stages. First, the data were screened for missing values and invalid response patterns. A sensitivity power analysis was then conducted using G^*^Power 3.1 to evaluate the statistical power afforded by the achieved sample size. With a sample of 1,236 participants, an alpha level of 0.05, and statistical power of 0.95, the analysis indicated that the study was capable of detecting an effect size as small as *f*^2^ = 0.018, suggesting that the sample size was adequate for the planned regression analyses. Second, the psychometric properties of the study measures were evaluated. The internal consistency of the multi-item psychometric measures was assessed using Cronbach's alpha coefficients. Confirmatory factor analyses (CFAs) were also conducted to examine their construct validity. Model fit was evaluated using the chi-square statistic, comparative fit index (CFI), Tucker–Lewis index (TLI), root mean square error of approximation (RMSEA), and standardized root mean square residual (SRMR). Third, the potential influence of common method bias was assessed using both exploratory and confirmatory procedures. Harman's single-factor test was first conducted as a preliminary diagnostic assessment. A CFA-based comparison was subsequently performed between a single-factor model, in which all measurement items were specified to load onto a single common factor, and the theoretically specified four-factor model. The difference in model fit between the two models was also examined. Fourth, descriptive statistics, including means and standard deviations, were calculated for the principal study variables. Pearson correlation analyses were then performed to examine the bivariate associations among physical activity, psychological resilience, cognitive reappraisal, and music performance anxiety. Fifth, a series of regression models was estimated to examine the individual paths specified in the proposed mediation model while controlling for gender, age, academic year, and years of musical training. The mediation analyses were conducted using Model 6 of the PROCESS macro in SPSS 26.0, following Hayes' procedure for serial mediation analysis (Hayes, 2017). Finally, the direct and indirect effects were evaluated using bootstrap resampling with 5,000 resamples. Ninety-five per cent bootstrap confidence intervals were generated for the specific indirect effect through psychological resilience, the specific indirect effect through cognitive reappraisal, and the sequential indirect effect through psychological resilience and cognitive reappraisal. An indirect effect was considered statistically significant when its 95% bootstrap confidence interval did not include zero (Zhao and Lynch, 2010).

## 5 Results

### 5.1 Common method bias test

To reduce the potential influence of common method bias, several procedural remedies were implemented during questionnaire design, including ensuring anonymity and confidentiality, clarifying the academic purpose of the study, reducing social desirability pressure, using clear and non-leading item wording, and randomly presenting items to minimize consistency bias. Harman's single-factor test was first conducted as a preliminary diagnostic assessment. The first unrotated factor accounted for 28.61% of the total variance, which was below the commonly adopted threshold of 40%. To further assess potential common method bias, confirmatory factor analysis was conducted. The single-factor model showed poor fit to the data, χ^2^(495) = 6184.43, CFI = 0.628, TLI = 0.603, RMSEA = 0.096, and SRMR = 0.105. By contrast, the theoretically specified four-factor model demonstrated good fit, χ^2^(489) = 983.89, CFI = 0.968, TLI = 0.965, RMSEA = 0.029, and SRMR = 0.027. The four-factor model fitted the data significantly better than the single-factor model, Δχ^2^ = 5200.54, Δdf = 6, *p* < 0.001. Taken together, these results indicated that no serious common method bias was detected in the present study.

### 5.2 Correlation analysis

As shown in Table 1, descriptive statistics and Pearson correlation analyses were conducted for the main study variables. Physical activity was significantly and positively correlated with psychological resilience (*r* = 0.355, *p* < 0.01) and cognitive reappraisal (*r* = 0.316, *p* < 0.01). Psychological resilience was also significantly and positively correlated with cognitive reappraisal (*r* = 0.342, *p* < 0.01). In contrast, music performance anxiety was significantly and negatively correlated with physical activity (*r* = −0.374, *p* < 0.01), psychological resilience (*r* = −0.398, *p* < 0.01), and cognitive reappraisal (*r* = −0.385, *p* < 0.01).

**Table 1:** Correlation analysis.

Variable	Mean	*SD*	1	2	3	4
1. PA	15.59	10.14	1			
2. PR	23.74	8.90	0.355^**^	1		
3. CR	26.62	6.67	0.316^**^	0.342^**^	1	
4. MPA	36.36	15.66	−0.374^**^	−0.398^**^	−0.385^**^	1

PA, physical activity; PR, psychological resilience; CR, cognitive reappraisal; MPA, music performance anxiety. ^**^p < 0.01.

### 5.3 Mediation analysis

As shown in Table 2, a series of regression analyses were conducted to examine the paths in the proposed mediation model while controlling for gender, age, academic year, and years of music study. Physical activity was significantly and positively associated with psychological resilience (*B* = 0.308, *SE* = 0.023, *t* = 13.193, *p* < 0.01). In the model predicting cognitive reappraisal, physical activity (*B* = 0.146, *SE* = 0.018, *t* = 7.932, *p* < 0.01) and psychological resilience (*B* = 0.195, *SE* = 0.021, *t* = 9.284, *p* < 0.01) were both significantly and positively associated with cognitive reappraisal. In the model without the proposed mediators, physical activity was significantly and negatively associated with music performance anxiety (*B* = −0.574, *SE* = 0.041, *t* = −14.029, *p* < 0.01). After psychological resilience and cognitive reappraisal were included in the full model, the negative association between physical activity and music performance anxiety remained significant but decreased in magnitude (*B* = −0.328, *SE* = 0.041, *t* = −7.910, *p* < 0.01). Psychological resilience (*B* = −0.429, *SE* = 0.048, *t* = −9.001, *p* < 0.01) and cognitive reappraisal (*B* = −0.555, *SE* = 0.063, *t* = −8.858, *p* < 0.01) were also significantly and negatively associated with music performance anxiety.

**Table 2:** Mediating effect.

Variable	PR			CR			MPA			MPA		
	*B*	*SE*	*t*	*B*	*SE*	*t*	*B*	*SE*	*t*	*B*	*SE*	*t*
Gender	0.732	0.473	1.548	0.361	0.349	1.036	0.175	0.828	0.211	0.769	0.766	1.004
Age	0.124	0.323	0.385	−0.132	0.238	−0.557	0.25	0.565	0.442	0.243	0.522	0.466
Grade	−0.342	0.415	−0.824	0.134	0.306	0.438	0.179	0.726	0.246	0.069	0.671	0.103
Yms	0.495^*^	0.232	2.135	0.232	0.171	1.357	−0.299	0.406	−0.737	0.096	0.376	0.255
PA	0.308^**^	0.023	13.193	0.146^**^	0.018	7.932	−0.574^**^	0.041	−14.029	−0.328^**^	0.041	−7.91
PR				0.195^**^	0.021	9.284				−0.429^**^	0.048	−9.001
CR										−0.555^**^	0.063	−8.858
*R* ^2^	0.132			0.163			0.141			0.268		
Adjust *R*^2^	0.128			0.158			0.137			0.264		
*F*	*F*_(5, 1230)_ = 37.276			*F*_(6, 1229)_ = 39.760			*F*_(5, 1230)_ = 40.255			*F*_(7, 1228)_ = 64.339		

PA, physical activity; PR, psychological resilience; CR, cognitive reappraisal; MPA, music performance anxiety; Yms, years of music study. ^*^p < 0.05, ^**^p < 0.01.

As shown in Table 3, the mediating effects of psychological resilience and cognitive reappraisal were further examined using a bootstrap procedure with 5,000 resamples. The 95% bootstrap confidence intervals for all three indirect effects did not include zero, indicating that each indirect pathway was statistically significant. Specifically, psychological resilience significantly mediated the association between physical activity and music performance anxiety, with an indirect effect of −0.132 (Boot SE = 0.018, 95% *CI* [−0.170, −0.098]). Cognitive reappraisal also significantly mediated the association between physical activity and music performance anxiety, with an indirect effect of −0.081 (Boot SE = 0.014, 95% *CI* [−0.109, −0.056]). Moreover, the sequential indirect pathway through psychological resilience and cognitive reappraisal was significant, with an indirect effect of −0.033 (Boot SE = 0.006, 95% *CI* [−0.045, −0.023]). These findings indicate that physical activity was indirectly associated with lower music performance anxiety through higher psychological resilience, higher cognitive reappraisal, and the sequential pathway from psychological resilience to cognitive reappraisal.

**Table 3:** Bootstrap method for mediating effect testing.

Path	Effect	Boot SE	BootLLCI	BootULCI
PA ⇒ PR ⇒ MPA	−0.132	0.018	−0.170	−0.098
PA ⇒ CR ⇒ MPA	−0.081	0.014	−0.109	−0.056
PA ⇒ PR ⇒ CR ⇒ MPA	−0.033	0.006	−0.045	−0.023

PA, physical activity; PR, psychological resilience; CR, cognitive reappraisal; MPA, music performance anxiety.

## 6 Discussion

This study examined the relationships among physical activity, psychological resilience, cognitive reappraisal, and music performance anxiety. The results showed that physical activity was associated with lower music performance anxiety. Psychological resilience and cognitive reappraisal each formed a significant specific indirect pathway, and the chain indirect pathway through both variables was also supported. The direct association between physical activity and music performance anxiety remained significant after both mediators were included in the model, indicating that psychological resilience and cognitive reappraisal accounted for only part of this association. Among the three specific indirect pathways, the indirect effect through psychological resilience was relatively larger, followed by the indirect effect through cognitive reappraisal, whereas the chain indirect effect was relatively smaller. Overall, this pattern suggests that the association between physical activity and music performance anxiety may involve several closely related but functionally distinct psychological factors. Specifically, psychological resilience reflects a broader capacity to remain stable under performance pressure and recover from disruption, whereas cognitive reappraisal more specifically concerns how individuals interpret performance-related stress, physiological arousal, and the risk of mistakes. These factors may each contribute independently to the association, while also forming a theoretically specified chain indirect pathway from psychological resilience to cognitive reappraisal. Taken together, the findings support the involvement of multiple psychological pathways in explaining the association between physical activity and music performance anxiety.

### 6.1 The relationship between physical activity and music performance anxiety

The results indicated a significant negative association between physical activity and music performance anxiety among university music students (*B* = −0.574, *p* < 0.01), supporting H1. Specifically, a one-point increase in the physical activity score was associated with an average 0.574-point decrease in the music performance anxiety score. This finding is broadly consistent with several previous studies. Rocha et al. (2014) found that music students with higher levels of physical activity reported lower levels of music performance anxiety. Similarly, a pilot study among wind musicians showed that performance anxiety decreased following a single bout of acute high-intensity exercise (Blasco-Lafarga et al., 2022). In addition, a systematic review and meta-analysis of undergraduate populations found that physical activity interventions were effective in reducing anxiety, depression, and stress (Huang et al., 2024). Collectively, evidence from music students, performing musicians, and broader undergraduate populations provides support for the negative association observed in the present study.

The negative association between physical activity and music performance anxiety may be related to the physiological and cognitive responses involved in performance anxiety. Music performance anxiety is not simply an experience of nervousness; rather, it encompasses physiological arousal, concerns about mistakes and evaluative outcomes, and avoidance-related responses. These reactions may become particularly pronounced in performance situations characterized by public exposure, external evaluation, and uncertainty regarding the outcome (de Lima et al., 2024; Kinney et al., 2025). Guyon et al. (2020) noted that performers' autonomic arousal before going on stage is closely related to their appraisal of the performance situation as threatening. When responses such as increased heart rate and muscular tension are interpreted as signs of imminent loss of control or likely performance failure, anxiety may intensify further. Regular physical activity has been associated with improved autonomic regulation and greater tolerance of stress-related physiological arousal (Anderson and Shivakumar, 2013). Physical activity may therefore help music students manage pre-performance physiological responses more effectively and reduce the extent to which these responses contribute to their subjective experience of anxiety.

The present findings should also be considered in relation to the distinctive academic and health-related circumstances of university music students. The Fit to Perform study reported that, although music students do not necessarily fare worse than their age-matched peers across all health indicators, some of the cognitions, attitudes, and behaviors associated with sustained practice and performance may adversely affect their health (Araújo et al., 2017). Longitudinal evidence further suggests that physical and psychological difficulties involving pain, stress, and anxiety may emerge from the early stages of university music education (Rosset et al., 2022). Music students are therefore exposed to the combined demands of intensive practice, precise skill execution, and repeated public performance. Within this context, physical activity may be relevant not only as a means of promoting general health, but also as a way of managing the physical and psychological demands associated with prolonged training and performance. The present study extends the limited evidence in this area by confirming, in a large sample, that physical activity is negatively associated with music performance anxiety. Nevertheless, because the study employed a cross-sectional design, the findings cannot establish that physical activity reduces music performance anxiety. Future research should use longitudinal or intervention designs and distinguish more carefully between different types, intensities, frequencies, and durations of physical activity to determine which patterns of activity are most consistently associated with music performance anxiety.

### 6.2 The mediating role of psychological resilience

The results showed that psychological resilience formed a significant independent indirect pathway between physical activity and music performance anxiety among university music students (indirect effect = −0.132, Boot SE = 0.018, 95% *CI* [−0.170, −0.098]), supporting H2. This finding is broadly consistent with the existing evidence, which suggests that physical activity is associated with better emotional well-being and stress coping and a greater capacity to cope with stress, while psychological resilience may represent an important factor linking physical activity to fewer negative psychological outcomes Lin et al., (2024). This pathway is particularly relevant in the context of music performance. Music performance anxiety does not arise solely from general academic or everyday stress; rather, it occurs in situations characterized by public exposure, immediate evaluation, and the execution of highly precise skills (Dobos et al., 2019). Performers are required to maintain motor control, retrieve memorized material, and sustain artistic expression while being observed by audiences or adjudicators. When anxiety disrupts attention or motor coordination, it may directly interfere with an ongoing performance (Twitchell et al., 2025; Du et al., 2025).

Psychological resilience may be associated with lower music performance anxiety because it reflects an individual's capacity to remain psychologically stable when confronted with pressure, mistakes, or uncertainty, and to refocus and continue performing after disruption. For music students, performance venues, acoustic conditions, instrument-related problems, stage disturbances, and other situational changes cannot always be fully controlled. At the same time, the evaluative nature of public performance may heighten concerns about mistakes and their consequences (Papageorgi et al., 2007; Šimunovič and Habe, 2025). Performers cannot simply interrupt or withdraw from the situation; instead, they must maintain control of their playing and sustain the intended musical expression under continuing pressure (Papageorgi et al., 2013). Individuals with greater psychological resilience may be more likely to view these demands as manageable challenges rather than uncontrollable threats and to recover their attention and performance focus more quickly following tension or error (Kalisch et al., 2015). Previous research has likewise reported that greater psychological resilience is associated with lower music performance anxiety (Du et al., 2025). The present findings therefore suggest that psychological resilience may help explain why students facing similar training and performance demands report different levels of music performance anxiety.

The positive association between physical activity and psychological resilience is also supported by previous research. Regular physical activity has been associated with more positive emotional experiences and greater self-efficacy (Li et al., 2022; McAuley and Blissmer, 2000). The goal persistence, self-regulation, and management of physical discomfort involved in exercise may also be related to a stronger capacity to cope with and recover from stress (Boat and Cooper, 2019; Salmon, 2001). (Lin et al. 2024) further showed that physical activity was positively associated with psychological resilience and that resilience formed a significant indirect pathway between physical activity and mental-health outcomes. The present study extends this pattern to music performance anxiety: music students reporting higher levels of physical activity also tended to report greater psychological resilience, which in turn was associated with lower music performance anxiety. Consistent with recent work in this field, psychological resilience is therefore better understood as a protective factor associated with performers' ability to maintain emotional stability, sustain attention, and continue performing under pressure, rather than as a single or deterministic causal mechanism (Twitchell et al., 2025; Du et al., 2025).

Overall, the findings support an indirect pathway linking physical activity, psychological resilience, and music performance anxiety. They further suggest that, within music education environments characterized by prolonged practice, public evaluation, and situational uncertainty, psychological resilience may be important for understanding individual differences in performance anxiety. Nevertheless, the cross-sectional data cannot establish the temporal ordering of the three variables or demonstrate that physical activity reduces music performance anxiety by increasing psychological resilience. Other factors, including self-efficacy, intrinsic motivation, and social support, may also contribute to the development and buffering of music performance anxiety (Ye, 2026). Future studies should therefore use longitudinal designs, experimental interventions, or multisource data to examine the role of psychological resilience in this relationship more rigorously.

### 6.3 The mediating role of cognitive reappraisal

The results further showed that cognitive reappraisal exerted a significant independent mediating effect in the relationship between physical activity and music performance anxiety among university students (indirect effect = −0.081, Boot SE = 0.014, 95% *CI* [−0.109, −0.056]), thereby supporting H3. This finding suggests that, in addition to resilience—a psychological resource more closely tied to stress recovery and adaptation—cognitive reappraisal, an emotion regulation strategy more directly concerned with the construction of situational meaning, may also represent an important pathway linking physical activity to music performance anxiety. Unlike generalized anxiety, music performance anxiety typically arises in contexts marked simultaneously by public exposure, immediate evaluation, and the execution of highly precise skills. Accordingly, how individuals interpret stage pressure and the accompanying physiological responses may play a crucial role in the development of anxiety (Guyon et al., 2020). Recent research has similarly emphasized that music performance anxiety is triggered not merely by physiological arousal itself, but is closely tied to individuals' cognitive appraisals of the performance situation, the consequences of mistakes, and their own internal states. When performers interpret responses such as accelerated heartbeat, trembling hands, or rapid breathing as signs of “losing control” or “impending failure,” such threat-based interpretations further amplify subjective anxiety and interfere with performance quality (Yang et al., 2025).

From this perspective, the mediating role of cognitive reappraisal carries clear theoretical significance. According to the process model of emotion regulation proposed by Gross and John (2003), cognitive reappraisal is an antecedent-focused emotion regulation strategy that involves altering subsequent emotional responses by reinterpreting the meaning of a situation or internal cues (Gross and John, 2003). For music students, the value of this strategy lies not in directly eliminating pre-performance arousal, but in helping them understand such responses in a less threatening and more adaptive way. For example, interpreting “nervousness” as a normal state of activation before performance, or viewing “physiological arousal” as the body's preparation for meeting the demands of a challenging task, may reduce catastrophic interpretations and excessive worry, thereby lowering subjective anxiety. Brooks' experimental work showed that reappraising anxiety as excitement can improve individuals' responses to stressful tasks (Brooks, 2014), which is also consistent with recent discussions in the music performance anxiety literature from the perspective of challenge–threat appraisal: more positive meaning-making helps reduce threat perception and promotes more stable task engagement (Guyon et al., 2020).

The present study further suggests that physical activity may be associated with this more adaptive mode of cognitive processing. Previous research has shown that regular physical activity not only contributes to improved cognitive functioning, particularly attentional control and executive functioning, but may also be linked to more effective emotion regulation processes (Smith et al., 2010). Giles et al for instance, found that habitual exercise was associated with better cognitive control and greater success in cognitive reappraisal (Giles et al., 2017). More recent work has likewise indicated that the frequency of cognitive reappraisal is associated with more positive affective experiences during exercise, and that such positive experiences are in turn linked to physical activity behavior (Gürdere et al., 2024). This suggests that the anxiety-reducing function of physical activity may not operate solely through general physiological regulation; more importantly, it may also be linked to individuals' psychological capacity for attentional control and the reinterpretation of discomforting experiences.

This explanation appears particularly applicable in the context of music performance. Physical activity itself is often accompanied by a certain degree of physiological arousal and task-related challenge, requiring individuals to continuously regulate how they interpret discomfort, effort, and stress signals, while repeatedly negotiating between “quitting” and “persisting” (Zhang et al., 2018). In this sense, exercise experience may provide individuals with a recurring everyday context in which to practice “how to interpret stress responses.” For example, they may come to view shortness of breath, muscle soreness, and similar reactions as normal aspects of the exercise process rather than as danger signals requiring immediate avoidance. Although such a cognitive processing pattern may not transfer directly to all performance situations, it at least offers a plausible theoretical framework for understanding why physical activity may be associated with higher levels of cognitive reappraisal and lower levels of music performance anxiety. Taken together, the findings support a significant specific indirect pathway linking physical activity to music performance anxiety through cognitive reappraisal. Higher physical activity was associated with greater use of cognitive reappraisal, which in turn was associated with lower anxiety, suggesting that how music students interpret performance-related stress and physiological arousal may partly account for this association.

### 6.4 The chain mediating roles of psychological resilience and cognitive reappraisal

The results showed that psychological resilience and cognitive reappraisal jointly exerted a significant chain mediating effect in the relationship between physical activity and music performance anxiety among university students (indirect effect = −0.033, Boot SE = 0.006, 95% *CI* [−0.045, −0.023]), thereby supporting H4. Given the large sample size, the statistical significance of this effect should be interpreted as evidence for the presence of the proposed psychological pathway rather than as indicating a substantial practical or clinical effect. Nevertheless, this finding suggests that the association between physical activity and lower music performance anxiety may partly reflect a sequential psychological process involving both stress-adaptation resources and the reappraisal of specific performance experiences. In the distinctive context of music performance, which is characterized by both public exposure and immediate evaluation, students need not only the capacity to withstand and recover from stress, but also the ability to interpret stage-related pressure and its accompanying physiological arousal in a less threatening manner. The chain mediation model therefore offers a more differentiated account of the psychological processes that may link physical activity with music performance anxiety.

Previous research has indicated that music performance anxiety is not triggered by a single factor, but rather involves multiple interrelated processes, including threat appraisal of the performance situation, anxiety over the consequences of mistakes, and heightened sensitivity to external evaluation (Twitchell et al., 2025). Theoretically, the present findings support an explanatory pathway in which resource stabilization precedes cognitive construction. Earlier research has typically conceptualized psychological resilience as a core psychological resource that helps individuals maintain emotional stability and adapt to stress (Fletcher and Sarkar, 2013), while cognitive reappraisal has often been treated as a relatively independent emotion regulation strategy (Gross, 2015). However, recent research has increasingly emphasized that the two are not entirely separate. Individuals with higher resilience are often better able to maintain emotional stability and cognitive openness under stress, and are therefore more likely to adopt positive and constructive modes of meaning-making. In other words, resilience may provide an essential resource base for the effective use of cognitive reappraisal (Fletcher and Sarkar, 2012; Kalisch et al., 2015; Tugade and Fredrickson, 2004). At the same time, recent studies have also suggested that resilience and cognitive reappraisal may not operate in isolation, but may instead exhibit a sequential relationship in the process of coping with stress, jointly relating to negative psychological outcomes (Liao and Hu, 2025). Other studies likewise indicate that highly resilient individuals typically possess stronger emotional recovery capacity and greater cognitive flexibility (Tugade and Fredrickson, 2004), making them less likely to rapidly fall into catastrophic or ruminative processing when stress arises and more likely to preserve the cognitive space needed to reinterpret the situation (Ursu and Măirean, 2022).

This is particularly important in the context of music performance. Music performance anxiety is often not a simple response to a single stimulus, but rather a dynamic process shaped by the interplay of threat appraisal, physiological arousal, and concerns about mistakes and social evaluation (Guyon et al., 2020; Kenny, 2011). Accordingly, when students lack sufficient resilience, even mild increases in heart rate, hand tension, or fluctuations in attention may be rapidly interpreted as signs of “losing control” or “certain failure,” thereby triggering ongoing cognitive worry and impaired performance (Osborne and Franklin, 2002). By contrast, higher resilience may enable individuals to retain a degree of emotional stability and perceived control in the initial impact of stress, thereby creating the conditions for the subsequent use of cognitive reappraisal. Cognitive reappraisal, in turn, may further help them interpret physiological arousal as part of task activation and view stage pressure as a manageable challenge rather than an unbearable threat.

Taken together, the present findings support a chain indirect pathway linking physical activity to music performance anxiety through psychological resilience and cognitive reappraisal. Previous studies have separately shown that physical activity is associated with lower music performance anxiety (Rocha et al., 2014), that psychological resilience mediates the relationship between physical activity and general mental-health outcomes (Lin et al., 2024) and is associated with lower music performance anxiety (Du et al., 2025), and that habitual physical activity is related to better cognitive control and more successful cognitive reappraisal (Giles et al., 2017). Although these studies provide an empirical basis for the individual associations included in the present model, the available evidence has largely remained dispersed across different research questions and analytical models. To our knowledge, previous studies have not examined these four variables together within an integrated model among university music students. A key contribution of the present study is its simultaneous examination of the specific indirect effects of psychological resilience and cognitive reappraisal, together with the chain indirect pathway operating through both variables. In doing so, the study integrates previously fragmented associations into a more process-oriented account. More specifically, physical activity was associated with a greater capacity to tolerate and recover from stress, which may provide the psychological conditions needed to maintain emotional stability and cognitive flexibility in high-pressure performance situations. This adaptive capacity may, in turn, be associated with a greater tendency to interpret stage-related pressure and physiological arousal in less threatening ways, thereby relating to lower music performance anxiety. These findings not only extend current understanding of the association between physical activity and music performance anxiety, but also provide a more specific account of the psychological processes that may contribute to differences in anxiety among music students facing similar performance demands.

## 7 Practical implications

The findings of the present study offer several practical implications for music teaching in higher education. First, within the reasonable scope of curriculum design, music programs may appropriately incorporate elements related to physical activity—for example, by offering foundational exercise courses for arts students or by integrating brief, optional physical activity sessions into rehearsal and training routines. Such low-cost, easily embedded activities may help enhance students' bodily awareness and strengthen their resource reserves for coping with physiological arousal. Second, music instruction may incorporate course designs oriented toward the cultivation of resilience. For instance, arranging small-scale stage presentations, introducing progressively challenging performance situations, or organizing constructive discussions following performance mistakes may provide students with manageable stress contexts in which to practice emotional recovery and stress coping. These strategies are not only well aligned with the contextual characteristics of music training, but also consistent with the resilience literature, which suggests that moderate stress can promote adaptation. Third, universities may use courses or workshops to guide students in learning a broader range of emotional and cognitive regulation strategies, such as alternative ways of interpreting physiological tension and stage-related stimuli, thereby supporting the development of more flexible cognitive processing patterns in highly evaluative situations. Taken together, integrating physical activity promotion, resilience cultivation, and emotion regulation training into the overall curricular structure of music education may help create a teaching environment that places greater emphasis on students' coordinated physical and psychological development, thereby providing more comprehensive support for music students facing highly demanding performance tasks.

## 8 Limitations and future directions

First, this study employed a cross-sectional design, which makes it difficult to establish the temporal ordering of the variables. Physical activity, resilience, and cognitive reappraisal may involve reciprocal or bidirectional relationships. Future research could adopt longitudinal designs, multi-wave follow-up surveys, or experimentally induced performance-stress paradigms to strengthen the rigor of causal inference.

Second, the sample was obtained through convenience sampling from accessible undergraduate music students at universities in several regions of China, which may have introduced selection bias. Because the study did not use probability-based or institution-stratified sampling, not all eligible music students had an equal chance of being included. Students who were more available during the survey period or more willing to participate may therefore have been overrepresented. Accordingly, the findings should be interpreted as reflecting associations within an accessible sample of Chinese university music students, rather than as evidence that can be generalized to all music majors in China. In addition, cultural characteristics such as highly evaluation-oriented educational environments and pervasive social comparison pressure may shape the expression of music performance anxiety, thereby further limiting the cross-cultural generalizability of the findings. Future studies should therefore employ broader and more systematically sampled populations and conduct cross-cultural or cross-educational-system comparisons to examine whether the patterns observed in the present study are robust across different cultural and educational contexts.

Third, although gender, age, academic year, and years of musical training were included as covariates, the present study did not assess or control for several other potentially relevant music-specific characteristics, such as instrument type, daily practice duration, the frequency and extent of public performance experience, competition or audition experience, and performance format. These factors may be associated with the performance demands experienced by students and with individual differences in music performance anxiety. Consequently, residual confounding cannot be ruled out, and the observed associations may partly reflect unmeasured differences in students' musical training and performance backgrounds. Future studies should collect more comprehensive information on music-specific training and performance characteristics and examine whether the proposed relationships remain robust after these factors are taken into account.

Fourth, all variables in this study were measured using self-report questionnaires. Although certain procedural controls were implemented during the research process to reduce common method bias, social desirability bias and recall bias may still have affected participants' responses. In addition, physical activity was measured using the PARS-3. This scale is a brief self-report instrument that primarily captures individuals' general physical activity patterns in terms of intensity, frequency, and duration. Although it has been widely used in Chinese research, its composite scoring method may still be limited in fully capturing the complexity of physical activity, its measurement precision, and individuals' actual total activity levels. The relevant findings should therefore be interpreted with caution. Future studies may integrate multisource data to achieve a more comprehensive assessment, such as physiological indicators (e.g., heart rate variability), objectively recorded physical activity data from wearable devices, and behavioral observations in performance settings, thereby enabling a more complete evaluation of physical activity and the related psychological processes.

Fifth, although the present study proposed a chain mediation model, several other psychological constructs closely related to music performance anxiety—such as perfectionism, self-efficacy, training culture, and social support—were not incorporated into the analytical framework. Future research could further develop multilevel or integrative structural models to expand the theoretical explanatory framework in this field.

Overall, future research should place greater emphasis on more rigorous tests of causality, the integration of multimodal data, more comprehensive assessment of music-specific training and performance characteristics, and cross-cultural comparisons, so as to develop a more complete model of the psychological processes associated with music performance anxiety and to provide more targeted pathways for educational intervention.

## 9 Conclusion

This study examined the relationships among physical activity, psychological resilience, cognitive reappraisal, and music performance anxiety and tested a chain mediation model integrating these variables. The findings showed that higher levels of physical activity were associated with lower music performance anxiety. Psychological resilience and cognitive reappraisal each mediated this association, and the chain indirect pathway through psychological resilience and cognitive reappraisal was also supported. Taken together, these findings suggest that the relationship between physical activity and music performance anxiety may involve both students' capacity to maintain or regain adaptive functioning under stress and their tendency to reinterpret performance-related experiences. By incorporating psychological resilience and cognitive reappraisal into the same analytical model, this study extends previous research that has primarily examined isolated relationships among these variables and highlights the important roles of stress adaptation and emotion regulation in understanding music performance anxiety. From a practical perspective, the findings provide preliminary reference for university music education when considering physical activity, resilience development, and cognitive reappraisal training in efforts to support students experiencing performance-related anxiety.

## Data Availability

The raw data supporting the conclusions of this article will be made available by the authors, without undue reservation.
